# Efficacy of Suprascapular Nerve Block and Pulsed Radiofrequency for Chronic Shoulder Pain in Parkinson’s Disease

**DOI:** 10.1155/prm/5852132

**Published:** 2026-05-29

**Authors:** Atak Karabacak, Tuğçe Gezer Karabacak, Banu Özen Barut

**Affiliations:** ^1^ Dr. Lütfi Kırdar City Hospital, Pain Clinic, Istanbul, Turkey; ^2^ Department of Physical Medicine and Rehabilitation, Marmara University Pendik Training and Research Hospital, Istanbul, Turkey, marmara.edu.tr; ^3^ Department of Neurology, Dr. Lütfi Kırdar City Hospital, Istanbul, Turkey

**Keywords:** pain management, Parkinson’s disease, pulsed radiofrequency, rigidity, shoulder pain, suprascapular nerve block

## Abstract

**Background:**

Shoulder pain is a frequent and clinically relevant nonmotor symptom in Parkinson’s disease (PD). Although suprascapular nerve block and pulsed radiofrequency (PRF) are used effectively for chronic shoulder pain in the general population, their therapeutic effects in patients with PD have not been clearly established.

**Objective:**

To assess the clinical efficacy of suprascapular nerve block combined with PRF in PD–related shoulder pain and its association with rigidity severity.

**Methods:**

This prospective, matched cohort study included 20 patients with PD and 20 age‐ and sex‐matched controls with chronic shoulder pain consistent with subacromial pain syndrome. All participants underwent ultrasound‐guided suprascapular nerve block combined with PRF. Pain intensity was assessed using the Numeric Rating Scale (NRS), and shoulder‐related pain and disability were evaluated using the Shoulder Pain and Disability Index (SPADI). Outcomes were recorded at baseline and during follow‐up. Associations between rigidity severity, levodopa equivalent daily dose (LEDD), and changes in clinical outcomes were analyzed using Spearman and partial correlation analyses.

**Results:**

Both groups demonstrated significant reductions in NRS and SPADI scores following the intervention (all *p* < 0.01). Although overall pain and disability improved, SPADI pain scores remained significantly higher in the PD group at follow‐up. Greater rigidity severity was consistently associated with reduced improvement in pain‐ and disability‐related outcomes, ranging from trend‐level to statistically significant correlations.

**Conclusion:**

Suprascapular nerve block combined with PRF provides meaningful pain relief in patients with PD and chronic shoulder pain. However, greater rigidity severity appears to limit clinical improvement, suggesting that disease‐specific motor features play an important role in shaping pain burden and response to peripheral interventions in PD. These findings highlight the need for multimodal pain management strategies addressing both peripheral and central disease–related factors.

## 1. Introduction

Shoulder pain and shoulder dysfunction represent clinically important but often underrecognized features of Parkinson’s disease (PD) [1, 2]. In some cases, shoulder pain or frozen shoulder may precede the diagnosis of PD [3]. Conservative approaches such as physiotherapy, exercise, hydrotherapy, and massage may be used in the management of shoulder pain [4]. In addition, the optimization of antiparkinsonian therapy may reduce shoulder pain in some patients [5]. Shoulder pain in PD may differ from that observed in the general population because of disease‐specific factors, including rigidity, bradykinesia, altered scapulothoracic biomechanics, and abnormal central pain processing [6]. These factors may influence both the clinical presentation of shoulder pain and the response to peripheral interventional therapies. Accordingly, evidence from non‐PD cohorts should be extrapolated to individuals with PD with caution.

The efficacy of suprascapular nerve block in the treatment of shoulder pain has been demonstrated in the literature [7]. In studies directly comparing pulsed radiofrequency (PRF) with local anesthetic injection alone, PRF has shown superior analgesic effects [8]. In addition, combined interventions using PRF together with steroid and local anesthetic have also been reported to be effective in the management of shoulder pain [9, 10]. However, the pathogenesis of pain in PD is likely to differ from that of the general population, and to the best of our knowledge, no previous study has specifically evaluated these interventions in patients with PD.

## 2. Methods

This study was designed as a prospective, comparative observational matched cohort study conducted at a tertiary care pain clinic between January 1, 2025, and January 1, 2026. Patients with PD who underwent suprascapular nerve intervention for chronic shoulder pain were prospectively enrolled based on predefined eligibility criteria.

### 2.1. Inclusion and Exclusion Criteria

Inclusion criteria for the PD group: Patients were eligible for inclusion in the PD group if they met the following criteria: age between 40 and 80 years; a diagnosis of idiopathic PD with Hoehn–Yahr Stages 1–3; and the presence of unilateral or bilateral shoulder pain persisting for at least 3 months. Shoulder pain was required to be clinically consistent with subacromial pain syndrome (SAPS), diagnosed based on established clinical criteria [11], including lateral or anterior shoulder pain exacerbated by arm elevation, relatively preserved passive range of motion, and positive findings on a combination of standardized clinical tests such as the Hawkins–Kennedy test, painful arc, and rotator cuff strength assessments.

To minimize potential confounding effects related to dopaminergic treatment adjustments on pain perception and motor performance, only patients with a stable antiparkinsonian medication regimen for at least 1 month prior to the intervention and throughout the 3‐month postprocedural follow‐up period were included.

Exclusion criteria were as follows: previous shoulder surgery, history of major shoulder trauma or radiologically confirmed fracture/instability, adhesive capsulitis, shoulder pain due to cervical pathology, evidence of systemic inflammatory disease (e.g., rheumatoid arthritis and psoriatic arthritis) or metabolic/infectious arthropathy, other significant neurologic or malignancy‐related pathologies that may explain shoulder pain, and patients unable to provide informed consent or to reliably complete study questionnaires were not referred to our clinic and, therefore, were not included in the study.

#### 2.1.1. Control Group

The control group consisted of patients with chronic shoulder pain who fulfilled the same clinical inclusion and exclusion criteria but had no diagnosis of PD. Control subjects were matched to the PD group for age, sex, and clinical shoulder pain presentation.

### 2.2. Evaluation Criteria and Timing

 Primary outcome measures were changes in pain intensity and shoulder‐related disability. Pain intensity was assessed using an 11‐point Numeric Rating Scale (NRS), ranging from 0 (*no pain*) to 10 (*worst possible pain*), as commonly used for measuring pain intensity [12] NRS scores were recorded at baseline (0 month) and at 1 and 3 months following the intervention. Shoulder pain and disability were evaluated using the Shoulder Pain and Disability Index (SPADI), an instrument originally developed to assess pain and functional impairment associated with shoulder pathology [13]. The Turkish version adapted and validated by Bumin et al. [14] was used in the present study. SPADI assessments were performed at baseline and at the 3‐month follow‐up visit.
 Secondary outcome measures included clinical rigidity severity and total daily levodopa equivalent daily dose (LEDD) in patients with PD. Rigidity was assessed using standardized clinical examination, and total LEDD was calculated according to established conversion formulas [15]. The relationships between rigidity severity, total LEDD, and changes in pain‐ and function‐related outcome measures were explored.

All outcome assessments were conducted at the same center using standardized protocols.

### 2.3. Ethics Approval

This study was reviewed and approved by the Institutional Scientific Research Ethics Committee in accordance with the Declaration of Helsinki and the International Conference on Harmonisation–Good Clinical Practice (ICH‐GCP) guidelines. Ethical approval was granted on December 25, 2024 (approval no: 2024/010.99/11/11).

### 2.4. Standardization of ON Period and Concurrent Treatments

All patients in the PD group were evaluated and underwent the intervention during the ON medication phase. No additional concurrent treatments, including analgesic dose adjustments, physical therapy or exercise programs, botulinum toxin injections, trigger point injections, or other interventional procedures, were permitted during the follow‐up period.

### 2.5. Indication and Target Shoulder

Interventions were performed unilaterally on the shoulder corresponding to the side of pain or, in cases of bilateral symptoms, the more painful side. All clinical assessments and outcome evaluations were standardized to the treated (more symptomatic) shoulder.

### 2.6. Interventional Method

All procedures were performed by the same interventional pain specialist under ultrasound guidance using a strict aseptic technique. Patients were positioned in the lateral decubitus position with the arm in neutral rotation. A high‐frequency linear ultrasound transducer, covered with a sterile sheath, was used to identify the suprascapular notch and the suprascapular nerve adjacent to the suprascapular artery.

Using an in‐plane approach, a 20‐gauge, 10‐cm radiofrequency cannula with a 10‐mm active tip was advanced into the perineural plane at the suprascapular notch under continuous sonographic visualization. Correct needle placement was confirmed by hydrodissection with 1‐2 mL of saline and by electrical stimulation. Sensory stimulation at 50 Hz elicited concordant shoulder paresthesia at ≤ 0.5 V, while motor stimulation at 2 Hz produced visible contractions of the supraspinatus and/or infraspinatus muscles at ≤ 0.6 V.

PRF treatment was then applied. Following negative aspiration and needle repositioning as required, a perineural block was administered incrementally to achieve circumferential spread around the nerve. The injectate consisted of a total volume of 6 mL, including 3 mL of 0.5% bupivacaine, 40 mg of triamcinolone acetonide, and 2 mL of 0.9% saline.

### 2.7. Equipment and PRF Parameters

Ultrasound guidance was provided using an Esaote MyLabSeven system (Esaote S.p.A., Genoa, Italy) with a high‐frequency linear probe.

PRF was delivered using the PRF mode of a Boston Scientific G4 RF generator. The PRF protocol consisted of a total application time of 360 s at 65 V, with a pulse frequency of 2 Hz and a pulse width of 20 ms. The maximum electrode tip temperature was limited to ≤ 42°C.

### 2.8. Motor Assessment

Motor features were assessed using routinely documented neurological examination findings obtained during standard clinical evaluations. Rigidity severity was assessed and recorded as part of routine clinical care by a single neurologist who was not involved in the interventional procedures, thereby minimizing inter‐rater variability.

Rigidity was graded on a 0–4 scale in accordance with the rigidity items of the Movement Disorder Society–Unified Parkinson’s Disease Rating Scale (MDS‐UPDRS) Part III [16]. For the purpose of this study, rigidity scores of the upper extremity corresponding to the painful shoulder were used in the primary analyses.

### 2.9. Levodopa Equivalent Dose Assessment

In patients with PD, antiparkinsonian medication regimens were reviewed, and the total daily LEDD was calculated for each patient using established conversion factors. Dose equivalency was determined according to the standardized method described by Tomlinson et al., which is widely used for reporting levodopa dose equivalency in PD research [15]. LEDD was evaluated as a potential covariate reflecting overall dopaminergic treatment intensity and disease‐related motor burden in exploratory analyses examining treatment response.

### 2.10. Statistical Analysis

Sample size was calculated a priori for the primary within‐group analysis in the PD cohort. For a paired‐samples *t*‐test with a two‐tailed alpha level of 0.05% and 80% power, the minimum required sample size was estimated to be 15 participants. Allowing for an anticipated dropout rate of 20%, the target sample size was set at 18 participants.

Continuous variables were evaluated for distributional characteristics and are presented as the mean ± standard deviation or median (interquartile range), as appropriate. Categorical variables were expressed as frequencies and percentages. Between‐group comparisons of baseline demographic characteristics were performed using the independent samples *t*‐test for continuous variables and Fisher’s exact test for categorical variables.

Within‐group changes in pain and shoulder‐related outcome measures (NRS and SPADI total, pain, and disability subscale scores) from baseline to follow‐up assessments were analyzed using paired statistical tests appropriate to data distribution. Between‐group comparisons of change scores (ΔNRS, ΔSPADI total score, and SPADI pain and disability subscales) were conducted using the Mann–Whitney *U* test due to the small sample size and the nonnormal distribution of clinical outcome measures.

Associations between rigidity severity and changes in pain‐related outcomes were examined using Spearman’s rank correlation analysis. To adjust for the potential confounding effect of dopaminergic treatment, partial correlation analyses were additionally performed controlling for LEDD. Relationships among outcome measures were assessed using correlation analyses to evaluate internal consistency and convergent validity.

All statistical tests were two‐tailed, and a *p* value < 0.05 was considered statistically significant. Statistical analyses were performed using IBM SPSS Statistics Version 31.0.

## 3. Results

There was no significant difference in age between the PD and control groups (67.3 ± 8.8 vs. 64.8 ± 11.0 years, *p* > 0.05).

The PD group consisted of 20 patients, of whom 13 were female (65%) and 7 were male (35%). The control group included 20 patients, with 14 females (70%) and 6 males (30%).

There was no statistically significant difference in sex distribution between patients with PD and the control group (Fisher’s exact test, *p* = 1.00).

In the PD group (*n* = 20), shoulder pain was located on the same side as the initial side of disease onset in 17 patients (85%).

In both the Parkinson and control groups, mean NRS scores decreased significantly from baseline to the 1‐month follow‐up, indicating an early treatment response (Parkinson: 7.6 ± 1.4–3.8 ± 2.2 and control: 7.8 ± 1.3–2.6 ± 1.8; both *p* < 0.001). Pain reduction was sustained at the 3‐month follow‐up in both groups, with mean NRS scores remaining significantly lower than baseline values (Parkinson: 4.2 ± 2.3 and control: 3.0 ± 1.9; both *p* < 0.001). Similarly, SPADI pain and disability scores showed significant improvement at month 3 compared with baseline in both groups (Parkinson: pain 63.7 ± 11.8–38.3 ± 19.3 and disability 45.8 ± 10.7–32.6 ± 17.6; control: pain 67.2 ± 7.7–28.9 ± 17.7 and disability 45.2 ± 7.8–23.8 ± 13.4; all *p* < 0.01) (Table 1).

**TABLE 1:** Within‐group changes in pain‐ and shoulder‐related outcomes.

Outcome	Group	Baseline	1 month	3 month	*p* value
NRS	Parkinson	7.6 ± 1.4	3.8 ± 2.2	4.2 ± 2.3	< 0.001
NRS	Control	7.8 ± 1.3	2.6 ± 1.8	3.0 ± 1.9	< 0.001
SPADI pain	Parkinson	63.7 ± 11.8	—	38.3 ± 19.3	< 0.01
SPADI pain	Control	67.2 ± 7.7	—	28.9 ± 17.7	< 0.01
SPADI disability	Parkinson	45.8 ± 10.7	—	32.6 ± 17.6	< 0.01
SPADI disability	Control	45.2 ± 7.8	—	23.8 ± 13.4	< 0.01

Between‐group comparisons of change scores were performed using the Mann–Whitney *U* test, with 20 participants in each group. No statistically significant difference was observed between groups in terms of ΔNRS (*p* = 0.088); however, the control group demonstrated a higher mean rank compared with the PD group (23.6 vs. 17.4). Similarly, changes in ΔSPADI total scores did not reach statistical significance between groups (*p* = 0.076).

In contrast, a statistically significant between‐group difference was found for the ΔSPADI pain subscale (*p* = 0.045), with the control group showing greater improvement than the PD group (mean rank: 24.2 vs. 16.8).

No significant between‐group difference was observed for the ΔSPADI disability subscale (*p* = 0.116); nevertheless, higher mean rank values were again noted in favor of the control group (23.4 vs. 17.6) (Table 2).

**TABLE 2:** Between‐group comparisons of change scores.

Outcome	Mean rank	*p* value
ΔNRS	23.6 vs. 17.4	0.088
ΔSPADI total	24.2 vs. 16.8	0.076
ΔSPADI pain	24.2 vs. 16.8	**0.045**
ΔSPADI disability	23.7 vs. 17.3	0.116

*Note:* The bold value indicates a statistically significant between‐group difference, defined as *p* < 0.05.

### 3.1. Correlation Between Rigidity Severity and Pain‐Related Outcomes

Spearman’s rank correlation analysis revealed a moderate negative association between rigidity severity and improvement in shoulder‐related disability as measured by ΔSPADI total scores (*ρ* = −0.43), which approached but did not reach statistical significance (*p* = 0.056). Similar trend‐level negative correlations were observed between rigidity severity and ΔSPADI pain, ΔSPADI disability, and ΔNRS scores, indicating a consistent pattern whereby greater rigidity severity was associated with reduced clinical improvement following intervention (Table 3).

**TABLE 3:** Spearman’s rank correlation analysis between rigidity severity and clinical improvement parameters.

Variable	Spearman *ρ*	*p* value (two‐tailed)
ΔSPADI pain (affected shoulder)	−0.408	0.074
ΔSPADI total	−0.434	0.056
ΔSPADI pain subscale	−0.382	0.097
ΔNRS	−0.431	0.058

### 3.2. Partial Correlation Analysis Adjusted for LEDD

Given the potential influence of dopaminergic treatment on pain perception, a partial correlation analysis was performed controlling for LEDD. After adjustment, rigidity severity demonstrated a significant negative association with improvement in SPADI total scores (partial *r* = −0.47 and *p* = 0.043), indicating that higher rigidity severity was independently associated with poorer improvement in shoulder‐related disability, irrespective of dopaminergic medication dose (Table 4).

**TABLE 4:** Partial correlation analysis between rigidity severity and clinical outcomes (partial correlation analyses were performed controlling for levodopa equivalent daily dose [LEDD]).

Outcome	Partial correlation coefficient (r)	*p* value
ΔSPADI disability	−0.394	0.095
ΔSPADI pain	−0.367	0.122
ΔSPADI total	−0.469	**0.043**
ΔNRS	−0.444	0.057

*Note:* Correlation coefficients (r) and two‐tailed *p* values are presented. The bold value indicates statistical significance.

Spearman correlation analysis demonstrated no significant association between LEDD and changes in SPADI total scores, SPADI pain or disability subscale scores, or NRS scores (all *p* > 0.05).

## 4. Discussion

In this exploratory study, we evaluated the clinical effects of suprascapular nerve block combined with PRF in patients with PD presenting with chronic shoulder pain consistent with SAPS and examined the relationship between rigidity severity and treatment response. The main findings were fourfold. First, both pain intensity and shoulder‐related disability, as measured by NRS and SPADI scores, decreased significantly following the intervention in the PD cohort, indicating a clinically meaningful treatment effect. Second, rigidity severity demonstrated a consistent negative association with improvements in pain‐ and disability‐related outcomes, ranging from trend‐level to statistically significant depending on the analytical approach. Third, despite overall improvement, SPADI pain scores remained significantly higher in patients with PD compared with the control group, suggesting disease‐specific factors influencing pain perception and modulation. Finally, in the majority of patients, the side of shoulder pain corresponded with the side of PD onset, supporting a close link between shoulder pain and asymmetric motor pathology.

The observed reductions in NRS and SPADI scores indicate that suprascapular nerve block and PRF can provide meaningful symptomatic relief in PD–related shoulder pain. To our knowledge, evidence specifically addressing the efficacy of suprascapular nerve–based interventions in PD is limited. Most prior studies on musculoskeletal pain in PD have focused on pharmacological management or generalized rehabilitation strategies, with little emphasis on targeted peripheral pain interventions [5, 17]. In this context, the present findings provide preliminary clinical evidence suggesting that combined suprascapular nerve block and PRF may have a role in the management of shoulder pain in PD.

An additional noteworthy observation was that, despite overall clinical improvement, SPADI pain scores remained significantly higher in patients with PD compared with the control group. This persistent difference suggests that shoulder pain in PD may not be fully explained by local mechanical or subacromial pathology alone. PD is associated with altered central pain processing, impaired descending inhibitory pathways, and abnormal sensory integration, all of which may contribute to heightened pain perception and reduced pain modulation [18, 19]. Shoulder dysfunction in PD is multifactorial and closely linked to disease severity, motor subtype, and rigidity‐related kinematic alterations rather than isolated structural lesions [20]. Such disease‐specific mechanisms may partially account for the sustained pain burden observed in the PD group despite comparable peripheral treatment.

The laterality of shoulder pain further supports this interpretation. In 85% of the patients, shoulder pain occurred on the same side as disease onset, a pattern recognized in PD [1, 21]. The presence of this asymmetry in our cohort reinforces the notion that shoulder pain is closely linked to the underlying asymmetric motor pathology, including rigidity and altered motor control, rather than representing a nonspecific musculoskeletal complaint. This observation adds contextual support to the disease‐specific nature of shoulder pain in PD.

Across multiple nonparametric analyses, greater rigidity severity was consistently associated with reduced improvement in SPADI total scores, SPADI pain and disability subscales, and NRS scores. Although unadjusted correlations did not uniformly reach conventional statistical significance, the direction and magnitude of the associations were consistent, suggesting a biologically meaningful relationship. Importantly, LEDD was not correlated with changes in pain or disability outcomes, and adjustment for dopaminergic medication dose did not materially alter the observed association between rigidity severity and treatment response. In a previous study, increased rigidity in PD was shown to be independently associated with higher pain frequency and greater pain‐related interference with work whereas tremor itself was not related to pain measures [22]. Consistent with this literature, our findings further demonstrate that rigidity severity is more closely linked to pain‐related outcomes than to functional disability, supporting a central role of rigidity‐related mechanisms in shaping pain burden and treatment response in PD.

Several pathophysiological mechanisms may underlie this relationship. Rigidity in PD is characterized by increased tonic muscle activity, altered shoulder biomechanics, and impaired scapulothoracic coordination [23]. These factors may perpetuate abnormal mechanical loading and sustained peripheral nociceptive input around the shoulder girdle, potentially limiting the analgesic and functional benefits of suprascapular nerve block and PRF, which primarily target peripheral nociceptive pathways. In this context, higher rigidity severity may reflect a motor phenotype that is inherently less responsive to localized peripheral interventions, despite technically adequate procedural delivery.

Interestingly, pain‐related outcomes appeared more sensitive to rigidity‐related differences than functional disability measures. This pattern is consistent with the notion that pain intensity may respond earlier or more directly to nociceptive modulation, whereas functional improvement is influenced by a broader array of factors, including motor impairment, activity avoidance, compensatory movement strategies, and fear of movement. The strong internal correlations observed between SPADI total scores and its subscales support the internal consistency of the outcome measures, while differences in statistical significance likely reflect limited statistical power rather than true discordance between pain and disability domains.

Several limitations should be acknowledged. While the sample size was adequate for the prespecified primary within‐group analysis in the PD cohort, it was limited for definitive between‐group comparisons; accordingly, the comparative findings should be interpreted with caution and considered exploratory. Rigidity assessment was based on a standardized clinical ordinal scale rather than instrumented biomechanical measurements; however, clinical rigidity scoring remains the standard approach in routine PD assessment and is widely used in clinical research [16, 24]. Furthermore, shoulder imaging was available and was used to support the eligibility assessment and exclusion of secondary pathologies, but imaging findings were not analyzed as study variables and no imaging‐based subgrouping was performed. Therefore, although the diagnosis of SAPS was clinically grounded, some heterogeneity in underlying shoulder pathology cannot be excluded. In addition, although PD–related motor features may have confounded the clinical assessment of shoulder pathology, the likelihood of substantial confounding was reduced by evaluating patients in the “ON” state, ensuring stable dopaminergic treatment, and limiting inclusion to Hoehn–Yahr Stages 1–3; nevertheless, residual diagnostic confounding cannot be completely excluded. Moreover, because suprascapular nerve block and PRF were applied as a combined intervention in all patients, the relative contribution of each component to the observed clinical improvement cannot be determined.

Despite these limitations, the present study offers clinically relevant insights. The findings suggest that while suprascapular nerve block and PRF can effectively reduce shoulder pain and disability in patients with PD, rigidity severity and disease‐specific pain mechanisms may limit the extent of clinical improvement. These results underscore the need for multimodal pain management strategies in PD, integrating peripheral interventions with approaches targeting rigidity, motor dysfunction, and central pain processing. Larger, prospective studies are warranted to confirm these observations and to further elucidate optimal treatment strategies for shoulder pain in this complex patient population.

## Funding

No funding was received for this manuscript.

## Conflicts of Interest

The authors declare no conflicts of interest.

## Data Availability

The data that support the findings of this study are available on request from the corresponding author. The data are not publicly available due to privacy or ethical restrictions.
